# Intravenous versus topical tranexamic acid in primary total hip replacement: A meta-analysis

**DOI:** 10.1097/MD.0000000000005573

**Published:** 2016-12-16

**Authors:** Pei Zhang, Yuan Liang, Pengtao Chen, Yongchao Fang, Jinshan He, Jingcheng Wang

**Affiliations:** a Dalian Medical University, Dalian, Liaoning; b Department of Orthopedics, Clinical Medical College of Yangzhou University, Subei People's Hospital, Yangzhou, Jiangsu, China.

**Keywords:** intravenous, meta-analysis, topical, total hip arthroplasty, tranexamic acid

## Abstract

**Background:**

As the prevalence of total hip arthroplasty (THA) is increasing, it is usually associated with considerable blood loss. Tranexamic acid (TXA) has been reported to reduce perioperative blood loss in hip joint arthroplasty. But the best route of TXA administration continues to be controversial. So, we conducted a meta-analysis that integrated all data from the 7 included trials to compare the effectiveness and safety of topical and intravenous TXA administration in primary THA. The endpoints assessed in this meta-analysis include the comparisons of total blood loss, postoperative hemoglobin decline, transfusion rates, the incidence rate of deep vein thrombosis (DVT), pulmonary embolisms (PE), and wound infection.

**Methods:**

Literature searches of PubMed, EMBASE, the Cochrane Library, the Chinese Biomedical Literature database, the CNKI database, and Wan Fang Data were performed up to August 30, 2016. Randomized controlled trials (RCTs) were included in our meta-analysis if they compared the efficiency and safety of intravenous versus topical administration of TXA in patients who underwent primary THA. The endpoints included the comparisons of total blood loss, postoperative hemoglobin decline, transfusion rates, the incidence rate of DVT, PE, and wound infection. A meta-analysis was performed following the guidelines of the Cochrane Reviewer's Handbook and the PRISMA statement. The pooling of data was carried out by using RevMan 5.3, Denmark.

**Results:**

Seven RCTs involving 964 patients met the inclusion criteria. Our meta-analysis indicated that there were no significant differences in the 2 groups in terms of total blood loss ([mean difference (MD) = −14.74, 95% confidence interval (CI): −89.21 to 59.74, *P* = 0.7], transfusion rates [RD = −0.02, 95% CI: −0.05 to 0.02, *P* = 0.39]; no significant differences were found regarding the incidence of adverse effects such as deep venous thrombosis [DVT] [RD = 0.00, 95% CI: −0.01 to 0.01, *P* = 1.00], PE [RD = 0.00, 95% CI: −0.01 to 0.01, *P* = 0.71], or wound infection [RD = −0.01, 95% CI: −0.06 to 0.04, *P* = 0.66]). The pooled results showed that the intravenous groups had a lower postoperative hemoglobin decline (MD = −0.47, 95% CI: −0.74 to −0.20, *P* = 0.0006). It was probably due to insufficient data and the varied reporting of outcomes. There was some inherent heterogeneity due to the small sample size of each primary study.

**Conclusion:**

The topical and intravenous administrations of TXA have a similar effect on the decrease of blood loss without an increased risk of complications (DVT, PE, and wound infection). Intravenous TXA administration may have a maximum efficacy. Topical TXA administration may be preferred in patients who with high risk of thromboembolic events. However, larger, high-quality RCTs are required to explore the optimal regimen, dosage, timing still in the future in order to recommend TXA widespread use in total joint arthroplasty.

## 1 Introduction

Total hip arthroplasty (THA) is an excellent surgical procedure for patients with end-stage hip diseases.^[1,2]^ By 2030, the demand for primary total hip arthroplasties is estimated to grow by 174% to 572,000.^[3]^ However, it is always accompanied by considerable blood loss, which may lead to acute anemia and a series of complications, increasing the risk of allogenic blood transfusion for patients.^[4,5]^ Allogenic blood transfusion could induce the patient's risk for adverse effects such as virus infection, immunologically mediated disease, cardiovascular dysfunction, and surgical site infection.^[6–8]^ However, allogeneic blood transfusion is an expensive resource that can induce more cost and prolonged hospital stays.^[9]^

Tranexamic acid (TXA) is a type of synthetic amino acid analog, which could block the lysine binding sites on plasminogen to inhibit activation of plasminogen and interfere with fibrinolysis.^[10]^ Now, TXA is widely used in surgical procedures such as prostate surgery, gynecological surgery, and cardiac surgery,^[11–13]^ and it is a growing knowledge that TXA would decrease the blood loss and transfusion rates. Many prospective randomized controlled and meta-analysis studies have proved that TXA applied topically or intravenously could decrease the blood loss and transfusion requirements without a high risk of complications, such as DVT, pulmonary embolisms (PE), or wound infection.^[14–21]^ The administrations of TXA have different kinds including intravenous (IV), orally, topically, or the combination of them. However, the optimal administration route of TXA remains controversial as the efficiency and safety of intravenous versus topical administration of TXA in THA was rarely reported.^[22–24]^ So we performed this meta-analysis that integrated all data from the 7 included randomized controlled trials (RCTs) to compare the efficiency and safety of intravenous versus topical administration of TXA in patients who underwent primary THA, including the comparisons of total blood loss, postoperative hemoglobin decline, transfusion rates, the incidence rate of DVT, PE, and wound infection.

## 2 Materials and methods

### 2.1 Literature search

**Figure 1:**
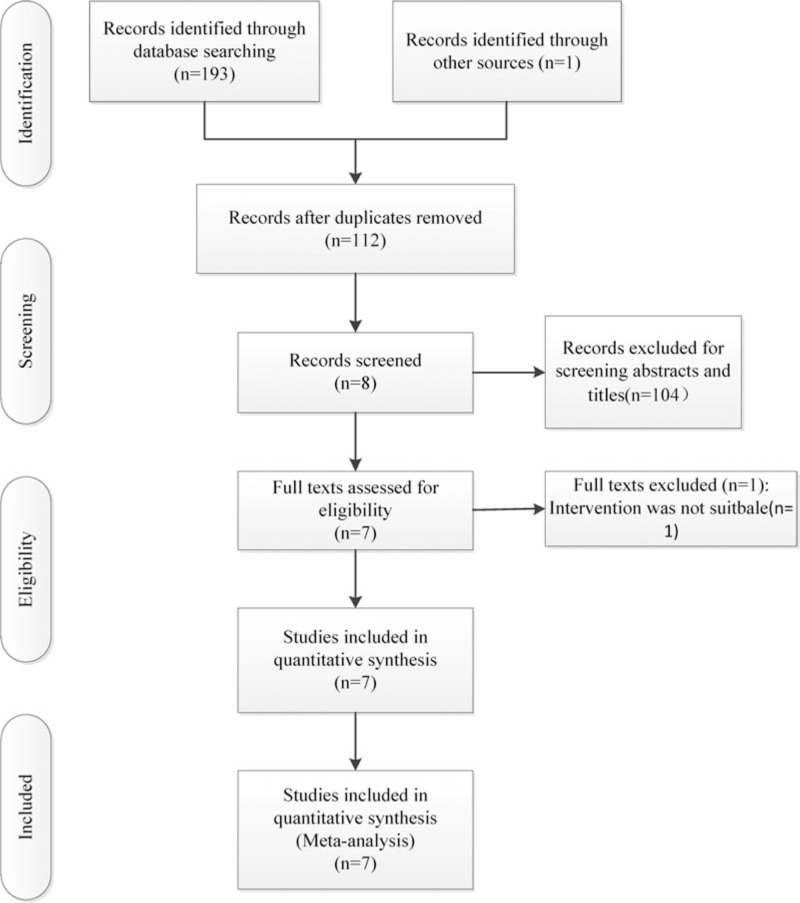
The flowchart of literature screening.

Literature searches of PubMed, EMBASE, the Cochrane Library, the Chinese Biomedical Literature database, the CNKI database, and Wan Fang Data were performed up to August 30, 2016. We also checked the references of the included literatures for potentially relevant studies. There were no language restrictions. The key words were used including “randomized controlled trials,” “tranexamic acid,” and “total hip replacement/arthroplasty.” We combined them with Boolean operators. The search results were performed in Fig. 1.

### 2.2 Inclusion and exclusion criteria

Trials could be eligible for inclusion if they met the following criteria: RCTs involved the comparison of the efficiency and safety of intravenous versus topical administration of TXA in patients who underwent primary THA; studies included at least one of the outcome measures. Studies were excluded if studies with incomplete data; patients had received other strategy to prevent blood loss; and participants with severe cardiovascular dysfunction (such as myocardial infarction), a history of thromboembolic events (DVT or PE), clotting disorders, a known allergy to TXA.

### 2.3 Data extraction

Two investigators scanned the studies to extract data independently by using a predefined data extraction form. Disagreement was resolved by consulting another investigator. The following data were collected: first author names, published year, study type, sample size, mean age, anesthesia methods, TXA intervention, prosthesis type, prophylactic antithrombotic therapy, transfusion trigger, and follow-up. Surgical outcomes for the meta-analysis including total blood loss, postoperative hemoglobin decline, transfusion rates, postoperative complications: VT, PE, and wound infection.

### 2.4 Assessment of methodological quality

Two investigators independently assessed the quality of the RCTs according to the method in the Cochrane Reviewer's Handbook 5.1.0. The risk of bias of each study was assessed according to the Cochrane risk assessment scale that includes the following contents: details of the methods of random sequence generation, allocation concealment, blinding, incomplete outcome data, selective outcome reporting, and other sources of bias. Any different opinions were resolved by a third reviewer.

### 2.5 Data analysis and statistical methods

RevMan 5.3 was used to pool all the included data for analysis. For continuous outcomes, we calculated the mean differences (MDs) with 95% confidence intervals (CIs); and the risk differences (RDs) with 95% CIs were calculated for dichotomous data. For the heterogeneity between studies, we pooled the data using the fixed-effect models or random-effect models. Statistical heterogeneity was assessed using the value of *P* and *I*^2^. When *I*^2^ > 50%, *P* < 0.1, which represented significant heterogeneity between studies, a random-effect model was applied. Otherwise, a fixed-effect model was used. If necessary, sensitivity analysis was conducted to identify the origins of the significant heterogeneity. Publication bias and meta-regression were not assessable in current meta-analysis, because test for funnel plot asymmetry and meta-regression are generally performed only when at least 10 studies are included in the meta-analysis. There were just 7 studies in our meta-analysis; thus, tests for asymmetry and meta-regression were not performed.

## 3 Results

### 3.1 Search result

A total of 112 potentially relevant references preliminarily reviewed. By scanning the titles and abstracts, 104 studies were excluded from analysis. After full texts assessed for eligibility, a study was excluded on the basis of inappropriate intervention. Finally, 7 RCTs published between 2014 and 2016 were included. The intravenous groups included 484 participants, and 480 patients in the topical groups.

### 3.2 Study characteristics and quality appraisal

**Figure 2:**
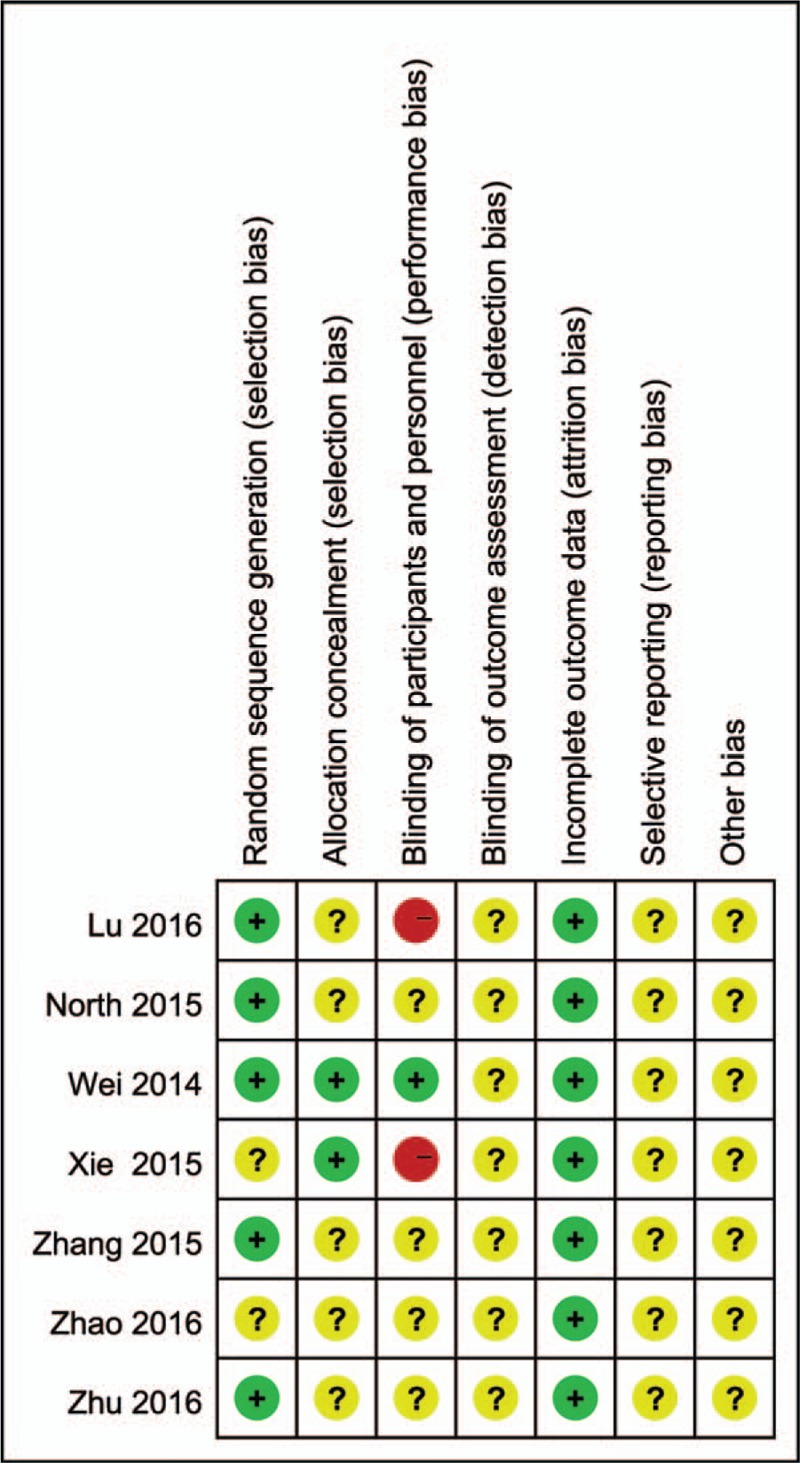
Methodological quality of the randomized controlled trials.

The characteristics of the included studies were shown in Table 1. The quality of the included RCTs is shown in Fig. 2. All of the studies reported clear inclusion and exclusion criteria. Six^[24–29]^ of the included RCTs reported that the randomization algorithm was generated from blinded biostatistician or a computer. Two of RCTs^[26,28]^ reported that the allocation concealment was achieved by opaque sealed envelopes. Only 1 RCT^[28]^ provided the information of double blinding. Binding of outcome assessment was not reported in the all studies. All of RCTs reported with complete outcome data. And only 1 RCT^[24]^ demonstrated intent-to-treatment analysis; therefore, the potential for type II statistical error would influence the results.

**Table 1:**
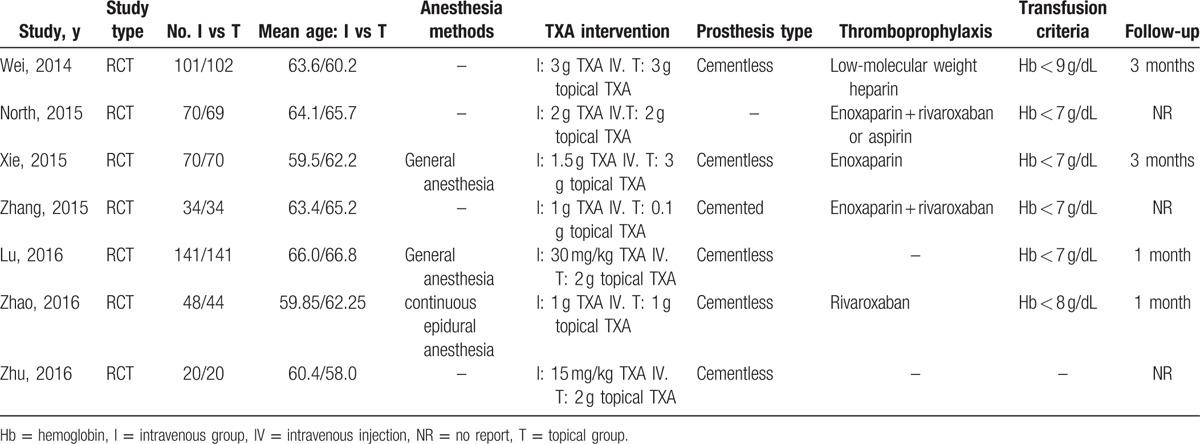
The characteristics of included studies.

### 3.3 Total blood loss

**Figure 3:**
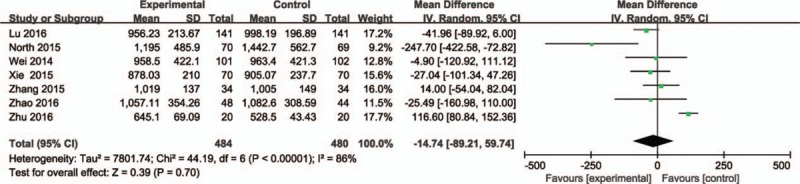
The comparisons of intravenous and topical tranexamic acid administration in total blood loss.

Seven studies (964 patients)^[24–30]^ compared the total blood loss. So, we included them as the data of the meta-analysis. There was significant heterogeneity between the studies (*P* < 0.00001; *I*^2^ = 86%); therefore, the random-effects model was used. The pooled results manifested that there was no significant difference between the intravenous and topical groups in terms of total blood loss (MD = −14.74, 95% CI: −89.21 to 59.74, *P* = 0.7; Fig. 3).

### 3.4 Postoperative hemoglobin decline

**Figure 4:**
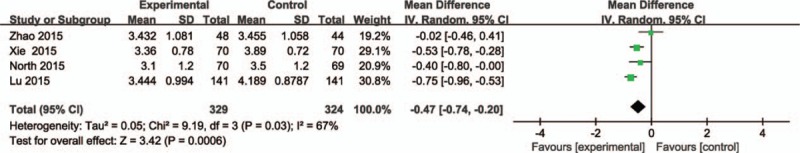
The comparisons of intravenous and topical tranexamic acid administration in postoperative hemoglobin decline.

Four articles (653 patients)^[24–26,30]^ reported the outcomes of postoperative hemoglobin decline. Heterogeneity was significant between the studies (*P* = 0.03; *I*^2^ = 67%); therefore, the random-effects model was performed. The pooled results showed that the intravenous groups had a lower postoperative hemoglobin decline (MD = −0.47, 95% CI: −0.74 to −0.20, *P* = 0.0006; Fig. 4).

### 3.5 Transfusion rate

**Figure 5:**
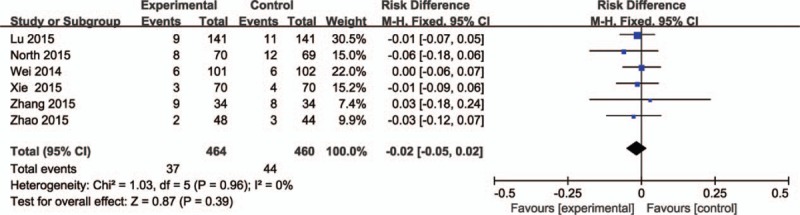
The comparisons of intravenous and topical tranexamic acid administration in transfusion rate.

Six studies (924 patients)^[24–28,30]^ compared the transfusion rate. No significant heterogeneity was detected between the studies (*P* = 0.96; *I*^2^ = 0%). Therefore, the fixed-effects model was used to do analysis. The results showed no significance difference between the groups regarding the transfusion rate (RD = −0.02, 95% CI: −0.05 to 0.02, *P* = 0.39; Fig. 5).

### 3.6 Deep vein thrombosis

**Figure 6:**
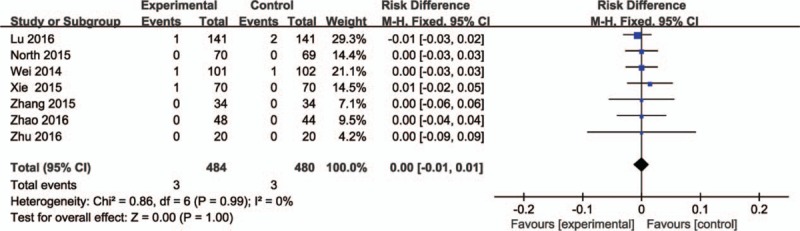
The comparisons of intravenous and topical tranexamic acid administration in incidence of deep vein thrombosis.

Seven literature sources (964 patients)^[24–30]^ reported the incidence of DVT. No significant heterogeneity was found (*P* = 0.99; *I*^2^ = 0%), so the fixed-effects model was used. It showed no significant difference between the groups (RD = 0.00, 95% CI: −0.01 to 0.01, *P* = 1.00; Fig. 6).

### 3.7 Pulmonary embolism

**Figure 7:**
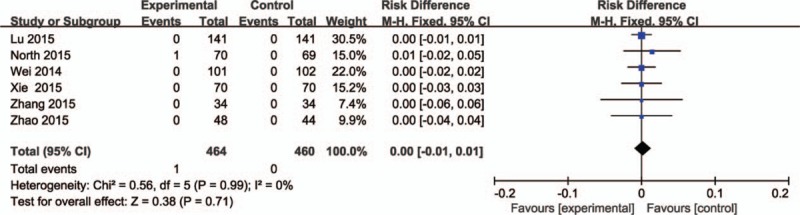
The comparisons of intravenous and topical tranexamic acid administration in incidence of pulmonary embolisms.

PE was reported in 6 included studies (924 patients).^[24–28,30]^ No significant heterogeneity was found (*P* = 0.99; *I*^2^ = 0%); therefore, the fixed-effects model was used. It manifested no significant difference between them (RD = 0.00, 95% CI: −0.01 to 0.01, *P* = 0.71; Fig. 7).

### 3.8 Wound infection

**Figure 8:**
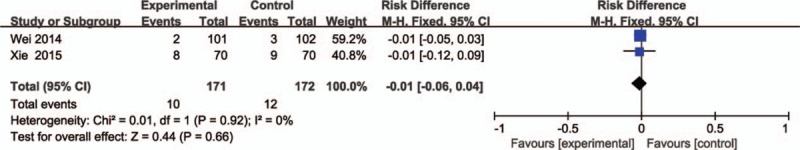
The comparisons of intravenous and topical tranexamic acid administration in incidence of wound infection.

Wound infection was reported in only 2 included studies (343 patients).^[26,28]^ No significant heterogeneity was found (*P* = 0.92; *I*^2^ = 0%); therefore, the fixed-effects model was used. It manifested no significant difference between them (RD = −0.01, 95% CI: −0.06 to 0.04, *P* = 0.66; Fig. 8).

## 4 Discussion

The most important finding of our meta-analysis was that there were no significant differences in the 2 administration methods of TXA. The topical and intravenous administrations of TXA have a similar effect in the decrease of blood loss without an increased risk of complications (DVT, PE, and wound infection). In present meta-analysis, we just included RCTs. It made our meta-analysis more credible.

The serum concentration of tissue plasminogen activator which activates fibrinolysis has a marked increase after surgery. TXA as a competitive inhibitor of plasminogen can block the activation, thus reducing total blood loss. The satisfactory results of a single intravenous dose of 15 mg/kg of TXA preoperatively or a topical use of TXA more than 2 g were proved.^[14,19–21]^ Ueno et al^[23]^ reported that there were no significant differences between topical TXA administration (2 g) and IV TXA administration (1 g) in terms of blood loss; therefore, a lower amount of TXA could be used to achieve appropriate outcomes in THA with IV TXA administration than with topical TXA administration. When IV TXA is given preoperatively, it is widely distributed throughout the extracellular and intracellular compartments and rapidly reaches maximum plasma concentration in 5 to 15 minutes.^[26,31]^ The local fibrinolysis can be inhibited at the initial stage as soon as surgery is commenced.^[26]^ Topical TXA administration has a higher drug concentration at the bleeding sites and a clearer view of the operation. In addition, topical TXA has a lower systemic absorption, which could minimize the occurrence of systemic complications,^[32]^ which is beneficial to the patients with a high risk of thromboembolic events following THA.

The primary endpoints of current meta-analysis were total blood loss, postoperative hemoglobin decline, and transfusion rates. Our results revealed that there was no significant difference between intravenous and topical groups in total blood loss. Significant heterogeneity was detected from the studies. It was probably due to the small sample size of each primary study, especially the study of Zhu et al.^[29]^ The heterogeneity between the studies had a great decrease when we removed it. However, the result did not change. Wind et al^[22]^ and North et al^[24]^ reported that blood loss was lower in the IV group. However, most of the studies comparing the 2 TXA administration methods, reported that there was no significant difference in blood loss.^[23,25,26,27,28,30]^ In the comparison of postoperative hemoglobin decline, the pooled results showed that the intravenous groups had a lower postoperative hemoglobin decline. It was consistent with the opinions of Wind et al^[22]^ and North et al.^[24]^ Although statistical difference was found in the postoperative hemoglobin decline, it was probably due to insufficient data.

Numerous studies have demonstrated that topical or IV administration of TXA leads to a statistically significant reduction in transfusion rates.^[22,33–36]^ No significant heterogeneity was detected in the studies. This meta-analysis showed that there was no significant difference in transfusion rates. The outcome had similar results with the included RCTs. Wind et al^[22]^ and Ueno et al^[23]^ also reported that there was no statically significant difference in transfusion rates between IV and topical administration of TXA through a retrospective study.

Considering that thrombotic complications could lead to severe results and even a death following THA, we should place great emphasis on them. Present meta-analysis showed no significant difference between the 2 groups in the incidence of DVT, PE. The outcome was consistent with results of previous studies.^[22,23,25–30]^ The CRASH-2 study which enrolling over 20,000 patients with more than 10,000 patients in each group, reported a decreased blood loss without any statically significant increase in vascular occlusive events.^[37]^ Therefore, we can infer that the different administration methods of TXA do not increase the risk of DVT and PE.

Another outcome of our meta-analysis was wound infection. Though the occurrence rate of wound infection is rare, it is very harmful to patients who could lead to a delayed wound healing, a lower outcome of joint function, even a revision surgery. In our meta-analysis, only 2 RCTs reported the events of wound infection. No significant heterogeneity was detected between the studies, and the result showed that there was no difference in the rate of wound infection. The present published data were not sufficient. The relation between wound infection and the application of TXA is still unclear; it is necessary to take further step to research it.

The anesthesia method was reported to have an effect on blood loss and transfusion requirement. Maurer et al^[38]^ and Basques et al^[39]^ reported that general anesthesia was associated with a significantly increased rate of adverse events and blood transfusion compared with spinal anesthesia. However, this conclusion has not been recognized; large sample size and multicenter studies were needed to confirm the conclusion.

The potential limitations in present study were followings: (first) some included trials excluding high-risk factors such as patients with a history of cardiovascular disease, the safety of TXA in high-risk patients should be explained cautiously; (second) the significant heterogeneity in total blood loss and postoperative hemoglobin decline, we could not conduct a subgroup analysis for the outcomes of total blood loss and postoperative hemoglobin decline due to the small sample size of each primary study; (third) in most of the studies, the methods of random sequence generation, the adequate concealment of allocation, the blinded assessments of the results were unclear. It could make a significant influence on the stability of the outcomes. (Fourth) The differences in surgical time, technique, and approaches as well as postoperative measures occur. (Fifth) The publication bias exists.

## 5 Conclusion

The topical and intravenous administrations of TXA have a similar effect in the decrease of blood loss without an increased risk of complications (DVT, PE, and wound infection). Intravenous TXA administration may have a maximum efficacy. Topical TXA administration may be preferred in patients who have high risk of thromboembolic events. However, more high-quality RCTs are required to explore the optimal regimen, dosage, timing still in the future in order to recommend TXA widespread use in THA.
